# Porcine Circovirus 2 Genotypes, Immunity and Vaccines: Multiple Genotypes but One Single Serotype

**DOI:** 10.3390/pathogens9121049

**Published:** 2020-12-14

**Authors:** Giovanni Franzo, Joaquim Segalés

**Affiliations:** 1 Department of Animal Medicine, Production and Health (MAPS), University of Padua, 35020 Legnaro (PD), Italy; 2 Centre de Recerca en Sanitat Animal (CReSA, IRTA-UAB), Universitat Autònoma de Barcelona, IRTA, 08193 Barcelona, Spain; joaquim.segales@irta.cat; 3 OIE Collaborating Centre for the Research and Control of Emerging and Re-Emerging Swine Diseases in Europe (IRTA-CReSA), 08193 Barcelona, Spain; 4 Department de Sanitat i Anatomia Animals, Facultat de Veterinària, Universitat Autònoma de Barcelona, 08193 Barcelona, Spain

**Keywords:** PCV-2, vaccine, genotypes, serotype, evolution, cross-protection

## Abstract

Identified for the first time in the 1990s, Porcine circovirus 2 (PCV-2) should not be considered an emerging virus anymore. Nevertheless, many aspects of its biology and epidemiology are still controversial. Particularly, its high evolutionary rate has caused the emergence of several variants and genotypes, alternating on the worldwide proscenium. The biological and practical implications of such heterogenicity are unfortunately largely unknown. The effectiveness of currently available vaccines against new genotypes that have emerged over time has been the topic of an intense debate and often inconclusive or contradictory results between experimental, field, and epidemiological studies have been gathered. The challenge in establishing an effective PCV-2 disease model, the peculiarities in experimental design and settings and the strains involved could justify the observed differences. The present work aims to summarize and critically review the available knowledge on PCV-2 genetic heterogeneity, immunity, and vaccine efficacy, organizing and harmonizing the available data from different sources, shedding light on this complex field and highlighting current knowledge gaps and future perspectives. So far, all vaccines in the market have shown great efficacy in reducing clinical signs associated to diseases caused by PCV-2, independently of the genotype present in the farm. Moreover, experimental data demonstrated the cross-protection of PCV-2a vaccines against the most widespread genotypes (PCV-2a, PCV-2b, and PCV-2d). Therefore, despite the significant number of genotypes described/proposed (PCV-2a to PCV-2i), it seems one single PCV-2 serotype would exist so far.

## 1. Introduction

Porcine circovirus 2 (PCV-2) is a member of the family *Circoviridae*, genus *Circovirus* [1]. It is featured by a circular single-stranded genome (ssDNA) of 1767–1768 nt where several open reading frames (ORFs) have been predicted in silico. However, just a few have been properly characterized. The ORF1 gene is located on the sense strand of the genome and produces several spliced variants, particularly Rep and Rep’, which are fundamental for viral replication. The ORF2 gene codes for the Cap protein that is the only constituent of the viral capsid; it is involved in the viral attachment and represents the main target of the host immune response [2]. Other proteins (coded by ORF3 to ORF6) seem to have a modulatory activity in the host cell pathways, signaling, and apoptosis [3,4,5,6].

PCV-2 was initially identified from pigs suffering from postweaning multisystemic wasting syndrome (PMWS), a novel disease described in the mid- 1990s [7,8]. PMWS is nowadays known as PCV-2-systemic disease (PCV-2-SD), and it comprises also what was initially described as PCV2-associated pneumonia and PCV-2-associated enteritis [9]. Other clinical-pathological conditions such as porcine dermatitis and nephropathy syndrome (PDNS), PCV-2-reproductive disease (PCV-2-RD) and PCV-2-subclinical infection (PCV-2-SI) have also been included into the scope of the collectively named porcine circovirus diseases (PCVD) [9]. Nevertheless, the definitive causative role of PCV-2 was debated at length because of the difficulty to experimentally reproduce PCV-2-SD by viral inoculation only. Additionally, retrospective studies proved PCV-2 presence well before the emergence of PCV-2-SD [10]. Indeed, PCV-2-SD is a typical example of a multifactorial disease, where other predisposing factors, most of those featuring modern intensive farming, must be in place to elicit overt clinical signs [11]. While some farms were able to live with the infection implementing adequate management and biosecurity, the most effective control measure was represented by the development of commercial vaccines, which became available from 2004 and 2006 onwards in Europe and North-America, respectively. These products led to a remarkable decrease of economic losses attributed to PCVD including PCV-2-SI [12]. Actually, vaccine efficacy represents one of the most consistent proofs in favor of the aetiological role of PCV-2 in PCVDs. PCV-2 vaccines are the single most-sold preventive product in porcine husbandry worldwide; nowadays, the vast majority of pigs and/or sows are vaccinated against PCV-2.

Nevertheless, in the last decade, a crescent concern has risen on the protection conferred against recently emerged genetic variants of PCV-2 [13]. The purpose of this work is to summarize and critically review the current knowledge on PCV-2 genetic variability and its relationship with vaccine efficacy, based on in silico, field, and experimental evidences.

## 2. Genotypes in PCV-2

Similarly to other ssDNA viruses, PCV-2 is featured by a high mutation rate (i.e., 10^−3^–10^−4^ substitution/site/year) [14,15], within the range typical of RNA viruses, which has led to the emergence of a plethora of variants over time. The accumulation of molecular epidemiology studies was mirrored by the implementation of several sub-species level classification schemes and nomenclatures, often based on subjective and/or conflicting criteria. A first effective harmonization attempt was made in 2008, when two major PCV-2 groups were defined based on nucleotide diversity cut-offs for ORF2 (3.5%) and complete genome (2.0%) [16]. These criteria were adopted by a European Project on PCVDs [17] and these two groups were proposed to be named as PCV-2a and PCV-2b. Based on the same criteria, PCV-2c was then identified from archived samples in Denmark [18]. Thereafter, the progressive increase in sequence availability and the discovery of new genetically divergent clades highlighted the limitations of such stringent genetic cut-offs and a new classification was proposed based on reference sequences and/or identification of marker positions, leading to the definition of 4 genotypes [19]. Currently, the most accepted scheme allowed defining eight genotypes (PCV2a to PCV2h), based on three criteria: maximum intra-genotype p-distance of 13% (calculated on the ORF2 gene), bootstrap support at the corresponding internal node higher than 70% and at least 15 available sequences [20]. Using such classification proposal, a new genotype PCV-2i has also been defined in the USA [21]. Therefore, the PCV-2 genetic scenario cannot be considered a static one, and new updates and changes on viral evolution are expected with a potential impact on genotype classification in the future.

Currently, PCV-2a, PCV-2b, and PCV-2d display a worldwide distribution while the other genotypes have been detected sporadically and limited evidence is present on their temporal persistence [20]. Of note, PCV-2c was considered extinguished or non-detectable for a long time, before being identified again in feral pigs in the Pantanal region of Brazil [22] and in domestic pig in China [23]. Similarly, other genotypes could be circulating, still undetected, in unexpected ecological niches (probably other Suidae species) and may serve as source of further genetic variability in the future. Nevertheless, the most important source of variation will be the domestic pig considering the abovementioned mutation rate of PCV-2.

Different epidemiological and phylodynamic studies revealed the occurrence of different genotype waves over time. PCV-2a was the most prevalent genotype in clinically affected pigs from 1996 to the early 2000s, after which PCV-2b predominated (“genotype shift”) and was associated with the appearance of a more severe clinical disease outbreaks [24,25,26]. Thereafter, a second “genotype shift” (from PCV-2b to PCV-2d) occurred globally [14,27] and has sometimes been reported in cases of vaccination failure [13,28]. However, the detection of other PCV-2 genotypes in vaccinated herds is not an unusual finding and the perception of a higher PCV-2d frequency in such herds could be biased by its rising global prevalence. Simultaneously, the presence of circulating recombinant forms (CRF) displaying comparable population dynamics and spreading routes to those of major genotypes has been demonstrated [14].

Although different PCV-2 genotypes have been historically identified sequentially, retrospective studies and molecular-clock based analyses proved their presence and co-circulation for decades. The actual reason behind the observed epidemiological patterns is not clear. A potential higher virulence of PCV-2b and PCV-2d strains has been suggested based on epidemiological patterns and some in vivo experimental data appear to support this hypothesis [29]. However, some other studies pointed out a similar virulence among genotypes [30,31]. Therefore, a putative differential virulence among genotypes is still to be demonstrated, although strain-specific differences could occur [32]. Importantly, virulence markers have been not defined for PCV-2 so far.

## 3. PCV-2 Immunological Cross-Reactivity

The pathogenesis of PCV2-SD depends on the final balance between the virus and the host immune response [33]. Different epitopic regions have been recognized both in the Rep and Cap proteins. The latter in particular is the main target of the host immunity and can elicit antibody and lymphocyte proliferative responses to PCV-2 [34,35]. Several linear or conformational epitopes have been also identified by PEPSCAN analysis, including amino acid residues 65–87, 117–131, 157–183, and 193–207 [36]. In addition, at least three conformational neutralizing epitopes, within residues 47–63, 165–200 and 230–233, have been described using chimeric PCV-1 and PCV-2 constructs [37]. Other linear epitopes (amino acids residues 156–162, 175–192, 195–202, and 231–233) have been recognized using monoclonal antibodies [38]. Finally, studies done to map immunogenic epitopes in the PCV-2 Cap protein have also demonstrated that several epitopes are shared between PCV-2 genotypes [37,38].

Accordingly, an overall immune cross-protection among PCV-2 genotypes exists and polyclonal antibodies are cross-reactive and cross-neutralizing [39]. Such protection breadth has been proven also under field conditions since sera from naturally infected pigs efficiently neutralized PCV-2 strains belonging to different genotypes and collected from different part of the word. However, a differential quantitative neutralization activity was identified, being the neutralization titre higher, on average, against PCV-2a than PCV-2b, which could justify the progressive spread of the latter genotype [40]. Noteworthy, these results were obtained with non-vaccinated pigs, so the higher antibodies against PCV-2a could not be attributed to a vaccination effect.

A more detailed picture emerged from studies using monoclonal antibodies. Saha et al. (2012a) detected the presence of common epitopes between PCV-2a and PCV-2b genotypes using monoclonal antibodies. However, the existence of genotype-specific antibodies was also demonstrated and some were able to recognize specific clusters within a genotype [41]. Single amino acid mutations were thereafter proven to alter the neutralization capability of some monoclonal antibodies [42,43].

Therefore, based on the observed evidence, while an overall cross-reactivity can safely be stated, some qualitative differences in the breadth and efficacy of immunity can be expected and involved in the PCV-2 epidemiologic patterns observed over time. The lower protection conferred by the immunity arisen against the prevalent genotypes and/or administered vaccines, based on PCV-2a, could have resulted in a fitness advantage of other genetic groups and, thus, their emergence in the world limelight. However, the generalized use of PCVC-2a vaccines all over the world also coincided with a “genotype shift” from PCV-2b to PCV-2d. Whether these vaccines are more effective on PCV-2b or a fast-evolving virus such as PCV-2 simply produced a novel genotype (PCV-2d) with better biological fitness is currently unknown.

Although less characterized, cell immunity plays a relevant role in protection against PCV-2 and the number of PCV-2 specific INFγ secreting cells (INFγ-SC) is inversely correlated to viral load and lesions [44,45]. Both Cap and Rep proteins are targeted by INFγ-SC, although a significant reactivity against Rep was reported in subjects with high viral titres and typical lesions, suggesting that high viral replication levels are necessary to elicit a significant response against non-structural proteins and that this immunity could be related in preventing the progression towards PCV-2-SD [46]. In silico epitope prediction revealed the presence of several potential cellular epitopes located both on Cap and Rep, some of those differing among circulating genotypes [47]. However, also in this case, experimental data showed that the cellular immunity induced by PCV-2a proteins is protective against PCV-2b challenge [46].

## 4. Vaccines, Genotypes and Evolution

PCV-2 vaccines became available in 2004 in Europe and 2006 in North America and have turned into the most implemented veterinary vaccines, contributing to remarkably decrease the impact of PCVD and the detrimental effects of the subclinical infection as well [48]. Moreover, a reduced viral excretion and susceptibility has been proven in vaccinated animals [49,50,51,52], leading to a reduction in reproductive ratio (R0) to 1.5 (95% CI 0.8–2.2) versus 5.1 (95% CI 2.5–8.2) under non-vaccinating conditions [53] and a decrease in overall PCV-2 circulation [54,55]. However, the implication of PCV-2 genetic and phenotypic diversity on vaccine efficacy is one of the most debated issues in the field, particularly after PCV-2d emergence. The vast majority of currently available vaccines are based on PCV-2a or its capsid protein, and the discovery of different neutralizing epitopes among genotypes or even ascribable to single mutations could justify the concern regarding a differential cross-protection and the presence of vaccine-escape variants [56]. Over time, a plethora of experimental studies has been performed using combinations of vaccine and challenge strains.

In all instances, PCV-2 vaccines appear to be protective, being able to effectively prevent clinical sign development, reduce viremia, viral shedding and lesions, and leading to the development of an effective humoral and cellular immune responses. No obvious evidence of a clinically significant differential cross-protection among PCV-2 strains could be proven, and all commercial vaccines seems effective in preventing the most severe outcomes of PCV-2 infection [48]. However, more subtle variations in virological or immunological parameters have sometimes pointed out higher protection conferred against homologous challenge than heterologous one. Opriessnig et al. (2013a) highlighted a stronger reduction in PCV-2b challenge viremia and shedding in pigs vaccinated with a PCV-1-PCV-2b chimera live vaccine compared to a PCV-1-PCV-2a one [57]. While the role of differential vaccine virus replication cannot be excluded in this case, higher PCV-2b titres were detected from nasal and faecal swabs in animals vaccinated with an inactivated PCV-1-PCV-2d chimeric vaccine compared with those immunized with the homologous PCV1-PCV2b one [58]. On the other hand, Park et al. (2019), while identifying a higher neutralization titre against PCV-2a in animals vaccinated with homologous commercial vaccines, found no differences in viremia after challenge with PCV-2a, PCV-2b, or PCV-2d strains, which could testify a role of cell-mediated immunity in terms of protection breadth [59]. Additionally, the challenge to establish an effective PCV-2-SD model, the peculiarities in experimental design and settings and the strains involved could justify the observed differences [60]. Nevertheless, the common conclusion, regardless of fine level differences, is the agreement on the adequate protection conferred by developed commercial and experimental vaccines against severe infection outcome, clinical disease and productive losses upon challenge with all evaluated PCV-2 genotypes [61,62,63,64], at least under experimental and controlled field trials.

The analysis of the epidemiological scenario and anecdotical reports, although difficult to be statistically evaluated, seems to tell a different story, being the observed genotype shift often linked to differential protection induced against the endemically circulating strains or available vaccines [14,65]. Accordingly, cross-protection against PCV-2a and PCV-2b was reported after natural infection, but with higher titre against PCV-2a [40]. Comparable results were recently reported in Korea, where a differential cross-protection among genotypes, and also between strains of the same genotype, was observed [66]. Reiner et al. (2015) found consistent evidence of a reduction in PCV-2a relative frequency in vaccinated herds compared to non-vaccinated ones, while the opposite was true for PCV-2b [67]. Epidemiological studies performed in the U.S. led essentially to the same results, with the PCV-2a positive samples originating mostly from non-vaccinated herds [65].

Taken as a whole, current evidence suggests that PCV-2 products are “leaky vaccines”, which can elicit adequate protection against clinical syndromes and reduce viral replication even when heterologous strains are involved. The presence of different epitopes, including neutralizing ones, is apparently balanced out by the efficient protective activity against shared ones. Nevertheless, viral infection and replication are not prevented [68] and under less optimal conditions (e.g., inaccurate vaccine administration, immunosuppression, declining maternally derived immunity, concomitant infections, etc.) the immunity could be less effective, magnifying these limitations. Accordingly, Jeong et al. (2015b) demonstrated the efficacy and equivalence of three commercial PCV-2a vaccines in solving a supposed episode of vaccine failure caused by PCV-2d under field conditions. Of note, one of the tested vaccines was already administered in the farm at the time of outbreak occurrence. Therefore a change in farm management and/or animal care could explain the apparent differential protection conferred by the same vaccine [61].

Vaccines have traditionally been considered much more resistant to pathogen evolution than antimicrobials [69]. Nevertheless, the role of vaccination in shaping viral evolution has been reported for different diseases affecting both animals and human beings. When immunity is not sterilizing, wild strains can circulate in a new “challenging” environment, made of less susceptible-immune hosts, adapting to it. Numerous examples are available of viruses that adapted to this new scenario by immuno-escape variants (Hepatitis B virus, avian Metapneumovirus, Infectious bronchitis virus), increase in virulence (Marek’s disease) or both (Infectious bursal disease virus) [70,71,72,73]. The aforementioned requirements for vaccine evolution and vaccine-induced pathogen replacement are present also for PCV-2 [39], which emphasizes the need of continuous surveillance and genotyping of this virus.

At the individual animal level, NGS-based studies demonstrated that, although the number of segregating sites was higher in non-vaccinating herds, the non-synonymous substitutions were more frequent in the vaccinated ones [56]. On the other hand, studies based on a large number of herds detected, besides to the aforementioned change in genotype prevalence, single nucleotide polymorphisms (SNPs) (also affecting epitopic regions) associated to vaccination status, suggesting an effect of vaccine-derived immunity on PCV-2 evolution [65,67]. The bioinformatic and phylodynamic analysis of PCV-2 epidemiological and evolutionary patterns at worldwide level reflects these pieces of evidence. PCV-2a was initially the most prevalent genotype, followed by PCV-2b and thereafter PCV-2d [14,27], although PCV-2b is still highly prevalent and the most prevalent one in certain countries/regions [74,75,76,77]. Analysis of selective pressures strength acting on PCV-2a highlighted a higher diversification tendency after vaccination introduction. Similarly, the viral population circulating in unvaccinated wild boar populations appears under lower selective pressures compared to domestic pigs [78].

Interestingly, PCV-2d was first detected retrospectively in Switzerland in 1998, but an increase in detection frequency of a sub-clade of PCV-2d has been reported in the years following vaccination introduction [14,79], which is indicative of a putative vaccine-induced replacement of a subset of genetic variants. Accordingly to this hypothesis, a statistically significant tendency of PCV-2a strains to mutate towards amino acids different from those of one commercial vaccine based on an inactivated PCV-2a virus, and identical to the amino acid profile of PCV-2d, was detected in at least 3 Cap sites after vaccination introduction, suggesting the appearance of vaccine-induced immuno-escaping evolutive trajectories [78]. Most interestingly, changes in each of these three amino acids (59-206-210) were experimentally demonstrated to impair the binding of monoclonal antibodies [36,41]. These data propose that the ancient PCV-2d strains had phenotypic features favouring them on a global scale in presence of vaccine immunity.

Although challenging to be consistently proven, the congruent pieces of evidence, ranging from individual to worldwide evolutionary scale, support the action of genotype-specific vaccine-induced immunity in progressively driving PCV-2 evolution, with likely detrimental effect on vaccine efficacy in the long term. If this path would ultimately lead to actual PCV-2a-based vaccine failure, is still a matter of discussion and definitively not yet proven.

## 5. The Usefulness of PCV-2 Genotyping and Emerging Genotypes

Genotypes are not a taxonomical level recognized by the International Committee on Taxonomy of Viruses (ICTV). Even if a certain consensus has been achieved on the PCV-2 nomenclature below the species level, this is essentially based on criteria chosen with the main aim of establishing a common and shared language. However, no guarantee exists that, depending on the research group involved, a variable nomenclature could be proposed and become accepted. As previously mentioned, despite remarkable efforts, the classification criteria are changing and evolving, largely because of the combination of viral evolution, increased sequencing activity and discovery of new strains.

Therefore, claims of full cross-protection based on genotype concept only are questionable, at first for the limits of any classification (i.e., the same PCV-2 strain included in the protection claim could be excluded according to a different or successive classification schemes and vice versa). Secondly, while the genotype concept has been widely accepted for historical reasons and is of practical utility in the framework of epidemiological studies, the PCV-2 genetics is much more nuanced. A clear overlap exists between the “within-genotype” and “between-genotypes” genetic distance [20]. In other words, because of the high evolutionary rate and recombination occurrence, the PCV2 genetic spectrum must be considered a non-stop, continuous concept; a formal concept that can biologically reflect the previously mentioned presence of strain-specific immunological features.

Finally, experimental data currently available on vaccine-induced protection has been obtained for the main circulating genotypes (i.e., PCV-2a, PCV-2b and PCV-2d), evaluated either as vaccine or challenge strains. Concerning the “minor” PCV-2 genotypes, the epidemiologic currently available evidence does not support any differential cross-protection conferred by existing vaccines. These variants have limited geographical distribution and have been detected only once or sporadically. If their genetic/antigenic features led to inadequate vaccine-induced protection, and thus to an evolutive advantage, a population size expansion, comparable to the one proposed for PCV-2d, should have been expected. However, their recent identification and limited data availability prevent any definitive conclusion and continued monitoring will be necessary to promptly react to a potentially changing scenario.

## 6. Conclusions and Future Perspectives

Like many aspects of PCV-2 infection and pathogenesis, the debate between vaccine efficacy versus vaccination failure is hard to be solved. Likely, a combination of both aspects, plus the presence of other factors, could negatively cooperate in the achievement of a “cut-off” of no or limited protection. While PCV-2a vaccines seem effective in protecting from clinical signs against all circulating genotypes under ideal vaccination conditions, different circumstances (e.g., improper vaccine administration or timing, animal health and herd management, etc.) could negatively affect the immunity and adequate protection cannot be reached in presence of strains with a reduced or limited cross-reactivity. Higher viral replication, infectious pressure, and even clinical signs could thus emerge, which would have not likely have occurred in presence of a homologous challenge. However, such scenario will be extremely difficult to ascertain in those farms with overt PCV-2-SD clinical signs in spite of vaccination, since there are multiple factors affecting vaccine efficacy as well as disease appearance in PCV-2 infected pigs.

Although proper herd management should always be considered a priority, the improvement and update of PCV-2 vaccines could likely contribute to the continuous improvement of animal health and performance. The inclusion of additional valency in vaccines has been suggested by several authors and recently implemented in some commercial vaccines, where a combination of recombinant chimeric PCV-1 expressing the porcine circovirus type PCV-2a and PCV-2b ORF2 genes was developed. The presence of PCV-2b, in addition to the direct improved protection against homologous challenge, could be beneficial against other genotypes, both because of its closer genetic distance than PCV-2a and because of a likely increase in protection breadth due to the presence of a combination of different epitopes. Recently, Opriessnig et al. (2020) demonstrated the comparable protection conferred by both PCV-2a and PCV-2b based vaccines after PCV-2d challenge and speculated that, since each of the vaccine viruses shares a discreet subset of B-cell and T-cell epitopes with the challenge virus, a bivalent vaccine containing both PCV2a and PCV2b components may have shown a higher degree of efficacy against a PCV-2d challenge compared to monovalent PCV-2 vaccines [80]. Accordingly, in silico prediction of T-cell epitopes also highlighted that bivalent vaccines may confer broader T cell epitope coverage against the evaluated genotypes (including PCV-2e) compared to the monovalent ones [47].

Comparable evidence emerges for other pathogens of veterinary interest, for which the combination of vaccines based on different genotypes has been traditionally considered a highly effective way to protect animals from new emerging variants of viral infectious diseases. This is the case of infectious bronchitis virus [81,82], which is featured by an even higher genetic and antigenic variability compared to PCV-2 [83]. Interestingly, a recent study evaluated the efficacy of an experimental vaccine obtained by DNA shuffling of the capsid genes of five genetically diverse PCV-2 subtypes (including PCV-2a, PCV-2b, PCV-2c, PCV-2d, and a divergent PCV-2a) [84]. Among the obtained clones, the one demonstrating the best performance had the vast majority of the amino acid signatures typical of PCV-2c and clustered phylogenetically within this genotype. Despite the remarkable genetic diversity, the developed vaccine provided the highest neutralization titre against PCV-2a, PCV-2b, and PCV-2d in vitro and the protection was confirmed in vivo using a PCV-2b and PCV-2d challenge viruses [84]. Therefore, the beneficial effect of different epitope combination rather than simple genetic resemblance is scientifically supported.

Limiting viral evolution and preventing further vaccine escaping variant emergence is another fundamental task for PCV-2 vaccinology monitoring. Also in this case, the use of a combination of different genotypes is likely to be helpful by creating a more heterogeneous immune response from which escaping through specific, single mutations would be less likely [73].

Although biologically sounded, these hypotheses are still in the field of speculation and dedicated studies should be performed both under experimental and field conditions. Particular care should be deserved to the analysis of viral evolution at the individual level, which represents the substratum for further evolution at epidemiolocal scale, benefitting of the crescent availability and accuracy of next generation sequencing technologies. Importantly, most of the currently available studies tested newly developed vaccines to unvaccinated subjects as controls, while a proper comparison and benefit evaluation should include animals vaccinated with currently available commercial vaccines as proper reference. Similarly, these new vaccines should be compared among each other, allowing to discriminate if the observed variations in virologic, immune, clinical, and productive parameters are ascribable to the vaccine itself or to the particular experimental settings.

In conclusion, PCV-2 is a fast-evolving virus that prompted the definition of a plethora of variants named genotypes. Although genotype variability is likely to increase in the future, current data indicates that cross-immunity is present among major genotypes (PCV-2a, PCV-2b, and PCV-2d) which, to date, guarantees vaccine induced protection by those products based on PCV-2a. Therefore, to date, we consider that the different genotypes of PCV-2 still represent one single viral serotype. In other words, PCV-2 genotypes conform a unique immunological variant with common antigenic properties so far covered by existing commercial vaccines.
